# Phase–Amplitude Coupling, Mental Health and Cognition: Implications for Adolescence

**DOI:** 10.3389/fnhum.2021.622313

**Published:** 2021-03-26

**Authors:** Dashiell D. Sacks, Paul E. Schwenn, Larisa T. McLoughlin, Jim Lagopoulos, Daniel F. Hermens

**Affiliations:** Thompson Institute, University of the Sunshine Coast, Sunshine Coast, QLD, Australia

**Keywords:** EEG, cross-frequency coupling, PAC, mental disorder, cognition, youth mental health, neurostimulation, DTI

## Abstract

Identifying biomarkers of developing mental disorder is crucial to improving early identification and treatment—a key strategy for reducing the burden of mental disorders. Cross-frequency coupling between two different frequencies of neural oscillations is one such promising measure, believed to reflect synchronization between local and global networks in the brain. Specifically, in adults phase–amplitude coupling (PAC) has been shown to be involved in a range of cognitive processes, including working and long-term memory, attention, language, and fluid intelligence. Evidence suggests that increased PAC mediates both temporary and lasting improvements in working memory elicited by transcranial direct-current stimulation and reductions in depressive symptoms after transcranial magnetic stimulation. Moreover, research has shown that abnormal patterns of PAC are associated with depression and schizophrenia in adults. PAC is believed to be closely related to cortico-cortico white matter (WM) microstructure, which is well established in the literature as a structural mechanism underlying mental health. Some cognitive findings have been replicated in adolescents and abnormal patterns of PAC have also been linked to ADHD in young people. However, currently most research has focused on cross-sectional adult samples. Whereas initial hypotheses suggested that PAC was a state-based measure due to an early focus on cognitive, task-based research, current evidence suggests that PAC has both state-based and stable components. Future longitudinal research focusing on PAC throughout adolescent development could further our understanding of the relationship between mental health and cognition and facilitate the development of new methods for the identification and treatment of youth mental health.

## Introduction

In Australia, mental disorders were estimated to have a direct economic cost of up to $51 billion and a further $130 billion cost as a result of diminished well-being during the 2018–2019 period (Productivity Commission, 2019). Mental health research and reform is currently focusing on adolescence as it is recognized as a critical period for the development of mental disorder (Paus, 2005; Hickie et al., 2013). More than 50% of mental disorders develop before 14 years of age and more than 75% before 24 years of age (Kessler et al., 2005). In Australia, 14% of those aged 4–17 years are evaluated as having a mental disorder in the prior 12 months (Lawrence et al., 2015). Young people frequently present with subthreshold, non-specific symptoms that are associated with significant, ongoing socio-occupational impairment (Lee et al., 2013; Iorfino et al., 2018) and an increased chance of developing discrete disorders (Fergusson et al., 2005; Iorfino et al., 2019). The rapid differential structural brain changes that occur during adolescence result in an increased risk of developing mental disorders during this period, as well as risk-taking behavior that can introduce various other risk factors for mental health (Andersen, 2003; Paus, 2005; Casey et al., 2010, 2011).

Traditional psychiatric approaches that focus on the identification and treatment of established mental disorders have limited application for developing psychopathology (Hickie et al., 2013). Thus, using neuroscience to investigate biomarkers related to youth mental health is critical to improving our understanding of emerging psychopathology and developing new methods of early identification and treatment. Phase–amplitude coupling (PAC), a type of cross-frequency coupling (CFC) between different frequencies of neural oscillation is a promising biomarker that may improve our understanding of mental health and cognition in adolescence. In this review article, we provide a brief overview of (1) CFC, specifically PAC in EEG research, (2) PAC in cognition/information processing, including memory, attention, language, and fluid intelligence, (3) PAC’s implications for neurostimulation and mental health, (4) PAC and structural connectivity, and (5) PAC developmentally. Finally, future directions for research are discussed.

## EEG and PAC

The EEG has been used to analyze the rhythmic, oscillatory activity of the human brain for nearly 100 years since Berger (1929) performed the first human EEG studies and discovered the alpha wave. Subsequently, an abundance of research has focused on delineating the properties and functions of individual frequency bands of various neural oscillations. The five most well-established frequency bands are: delta, theta, alpha, beta, and gamma, which have been implicated across a range of cognitive functions that vary according to context and associated region. For an overview, see Herrmann et al. (2016). Analysis of different frequencies independently aligns with the traditional perspective that the brain is comprised of phylogenetically distinct networks that operate at different frequencies. However, recent EEG research, in conjunction with electrocorticography (ECoG) and magnetoencephalography (MEG), suggests that there is a dynamic interplay between neural frequencies that reflects the integrative nature of cortical networks.

Cross-frequency coupling between two different frequencies of neural oscillations is hypothesized to facilitate communication between meso and microscales within regions, and synchronization and interaction between local and global networks in the brain inter-regionally (Canolty and Knight, 2010). Through use of source localization techniques and concurrent fMRI, studies are now able to provide greater spatial resolution to complement EEG analyses. A now common perspective is that oscillatory activity is hierarchical—the phase of slower oscillations modulates the amplitude, frequency, or phase of faster oscillations (Jerath et al., 2019). Recent efforts have focused on coupling between the phase of slower oscillatory activity and the amplitude of faster oscillatory activity (i.e., PAC). Importantly, it is now understood that PAC provides additional explanatory power beyond simple “neuronal communication through neuronal coherence” (Fries, 2005), with evidence suggesting that PAC may facilitate separate, spatially distributed cortical networks operating in parallel (van der Meij et al., 2012). Findings suggest that PAC is a fundamental neurological process that can potentially help us better understand information processing and mental health in the brain.

## PAC and Cognition

### PAC and Memory

One of the most researched areas in the context of PAC is memory. Numerous adult EEG studies have implicated theta–gamma PAC as a neural substrate of visual and auditory working memory (Axmacher et al., 2010; Kaminski et al., 2019). Theta–gamma PAC (one of the most prominent types of PAC) has been recorded within multiple brain regions, but specifically in the hippocampal region and prefrontal cortex during working memory tasks. These results have been replicated in adolescents. Theta–gamma PAC within frontal and temporal regions, as well as interregionally between frontal and posterior regions has also been implicated in the encoding and retrieval of items in long-term, declarative memory in adults (Friese et al., 2013; Lara et al., 2018; Köster et al., 2019).

One popular model of working memory suggests that individual gamma waves represent memory items, which are “nested” in theta, enabling the retention of multiple items. See Sauseng et al. (2019) for a review of evidence—pertinently, researchers were able to elicit temporary improvements in visual and verbal working memory using transcranial alternating current stimulation (tACS) to slow theta waves, theoretically enabling the nesting of an additional gamma wave (Vosskuhl et al., 2015; Wolinski et al., 2018).

### PAC, Language, Attention, and Intelligence

Other complex areas of cognition that have been associated with PAC in adults include language, attention, and fluid intelligence. PAC has been associated with linguistic processes such as verb generation (Doesburg et al., 2012), linguistic structure composition (Brennan and Martin, 2020), and language prediction (Wang et al., 2018). An oscillatory model of language is described by Murphy et al. across a series of studies (Murphy, 2016, 2018; Benítez-Burraco and Murphy, 2019).

Phase–amplitude coupling is also implicated in selective attention in adults (e.g., Doesburg et al., 2012; Saalmann et al., 2012). Gonzalez-Trejo et al. (2019) demonstrated differences between “car drivers” and “co-pilots” in PAC within the prefrontal cortex, frontal eye fields, primary motor cortex, and visual cortex during a simulated driving task. Mento et al. (2018) demonstrated that theta–beta coupling at Cz is associated with the temporal orienting of attention in 8–12-years-olds.

Chuderski (2016) criticized the methods used by previous research that reported a relationship between PAC and fluid intelligence (Pahor and Jaušovec, 2014) but highlighted that measures of intelligence are strongly correlated with working memory and thus likely associated with PAC. Gągol et al. (2018) identified that greater beta–gamma PAC coupling at rest and during a reasoning task was associated with increased fluid intelligence at individual sites across the brain, with greater strength at medial, frontal, and parietal electrodes. See Hyafil et al. (2015) for further discussion about the cognitive mechanisms of PAC. Further research is required to determine how findings in adults translate into adolescents. It is now well established that cognition and mental health are closely intertwined; consistent cognitive deficits are present across mental disorders in youth and relate directly to functioning and illness trajectory (Lee et al., 2013, 2018). Evidence increasingly suggests that PAC is a fundamental process underlying information processing.

## PAC, Neurostimulation, and Mental Health

### Neurostimulation

One of the most interesting developments in the PAC literature is that both temporary and lasting changes can be elicited by contemporary treatment modalities such as tACS (mentioned above), transcranial direct current stimulation (tDCS), and transcranial magnetic stimulation (TMS). Jones et al. (2020) demonstrated that combining tDCS with working memory training over 4 days resulted in significantly greater working memory improvements than working memory training alone in adults, which were mediated by greater inter-regional theta–gamma PAC strength between the prefrontal cortex and temporo-parietal regions. This is not only relevant to cognition, but also mental health.

Noda et al. (2017) analyzed resting-state PAC before and after a typical 2-week course (10 sessions) of repetitive TMS (rTMS) targeting the dorsolateral prefrontal cortex in adult patients with depression; rTMS was associated with improved scores on the Hamilton Rating Scale for Depression, Beck Depression Inventory and increased PAC at the C3 (left temporal) and T3 electrode site at rest. These findings suggest that decreased resting-state theta–gamma PAC may be a biomarker of poorer mental health (specifically depression) and that lasting changes in PAC may be a mechanism underlying TMS treatment. Notably, cross-frequency theta–gamma tACS protocols have been demonstrated to influence memory (Alekseichuk et al., 2016; Lara et al., 2018), cognitive control (Turi et al., 2020), and emotional-action control (Bramson et al., 2020). Alekseichuk et al. (2016) reported that theta–gamma tACS has a greater effect on working memory than theta tACS. If PAC is disrupted in mental disorder, then cross-frequency tACS may be a promising prospective treatment.

### Mental Disorder

Currently, research investigating the links between mental health and PAC is emerging. A number of studies have investigated the relationship between PAC and psychotic disorders. Barr et al. (2017) compared PAC between adults diagnosed with schizophrenia and healthy controls. Theta–gamma coupling within the prefrontal cortex was significantly reduced during a working memory task in the patient group compared to controls. Whereas increased theta–gamma coupling was associated with correct responses on a working memory task in the controls, there was no association between coupling and accuracy in the patient group. Theta–gamma coupling within the occipital region decreased with working memory load in controls, but there was no pattern in patients. Other studies (Won et al., 2018; Lee et al., 2019) have compared PAC at rest in healthy controls to young adults (*mean* age = 23.2 and *SD* = 4.9) with first-episode psychosis and adults with neuroleptic-naïve schizophrenia, respectively. Both studies identified increased theta–gamma PAC at rest within regions known to be associated with the default mode network (DMN) in patient groups compared to controls. These results suggest people with psychosis have dysfunctional hyperactivation of resting-state theta–gamma PAC within DMN-related brain regions, which may be a result of compensatory reallocation of cognitive resources due to dysfunction in the prefrontal cortex. Won et al. (2018) also reported that PAC better predicts patients with schizophrenia (with a classification accuracy of 92.5%) compared to power spectra analysis (which had 62.2% accuracy), suggesting that it is a promising neurophysiological marker of schizophrenia.

Another study compared theta–gamma PAC between young people with ADHD and healthy controls. The ADHD group demonstrated significantly reduced PAC within diffuse regions across the cortex during an arithmetic task compared to the controls. Deficits in PAC were associated with reduced performance on the arithmetic task. The authors hypothesized that reductions in PAC reflected deactivation of the DMN during the task, but failure to shift to attentional networks (Kim et al., 2016). Although further PAC research is required across a range of disorders, these early results suggest that PAC is associated with functional aspects of various mental disorders. Further detailed research is likely to provide a greater understanding of its mechanisms across cognition and mental health.

## PAC and Structural Connectivity

Currently, the exact mechanisms that underpin CFC are unknown. The EEG signal is believed to be predominantly generated by pyramidal cells in the cortex (for a full explanation of the EEG signal generation process, see Steriade et al., 1990; Kirschstein and Köhling, 2009). EEG functional connectivity is believed to be strongly influenced by white matter (WM) in myelinated axons—specifically cortico-cortical axons (Nunez et al., 2015). Through *in vivo* analysis of WM using contemporary MRI techniques such as diffusion tensor imaging (DTI) abnormalities in WM microstructure have been established across a range of mental disorders (Kanaan et al., 2005; Sexton et al., 2009; Heng et al., 2010). Although DTI resolution is too limited to create comprehensive maps of axon connectivity (Nunez et al., 2015), combining EEG and DTI may improve our understanding of how anatomical connectivity shapes functional connectivity (Cohen, 2011). Early research into alternative measures (cross-correlation and coherence) of EEG functional connectivity has identified associations with structural connectivity (Chu et al., 2015).

We are unaware of any studies to date that have implemented concurrent EEG and DTI to investigate how PAC is related to brain structure. Hawasli et al. (2016) hypothesized that WM and gray matter (GM) lesions in humans undergoing resection surgery for epilepsy or brain tumors would result in disruption to PAC. Contrary to their hypothesis, they reported that PAC was maintained after WM lesions, and GM lesions resulted in increased beta–gamma PAC in the resected area. Using concurrent DTI and EEG analyses may provide further insight into how the relationship between WM microstructure and PAC both within regions and across cortical networks affects information processing. Specifically, such research during adolescent brain development may help us better understand healthy and pathological neural development trajectories and thus lead to new methods of identification and treatment for mental disorder in youths.

## PAC and the Developing Adolescent Brain

The majority of PAC research to date has focused on cross-sectional adult samples. Although some recent studies investigating younger age groups have been completed (i.e., in memory, attention, and ADHD), there does not appear to have been any large-scale studies that have investigated PAC longitudinally, from a developmental perspective. As adolescence is known to be a critical period for the development of mental disorders, recent evidence that PAC is associated with mental health demonstrates a clear requirement for PAC research throughout this period. Understanding the emergence of pathology is critical for understanding the biological basis of mental illness. Because initial PAC research focused on task-based cognitive studies, some initial perspectives suggested that PAC is a transient, state-based measure, whereas other forms of CFC may be more steady and suited to longitudinal research. For example, amplitude–amplitude coupling (AAC) between lower frequency oscillations was suggested to be particularly suited to longitudinal research as it demonstrated trait like properties associated with motivational and emotional processes, as well as anxiety (Schutter and Knyazev, 2012). Knyazev et al. (2019) investigated AAC annually in 7–10-year-olds, finding that AAC appears to have non-linear growth trajectories with strong test–retest reliability and that higher AAC in cortical areas related to emotion, attention, and social cognition was related to introversion.

However, little is known about AAC as it has received relatively little attention in the literature compared to PAC. Recent evidence demonstrating that PAC is associated with mental health and cognition, including at rest and pre-post intervention, illustrates that PAC clearly has persistent, stable components equally suited to longitudinal research. This emerging research suggesting that PAC may be an important biomarker of mental health highlights the importance of longitudinal research investigating PAC throughout the rapid differential structural brain changes that occur during adolescence. Longitudinal research to determine whether PAC demonstrates similar non-linear growth trajectories to AAC and how these are associated with adolescent cognitive development and mental health may be crucial to better understanding how PAC research can contribute to better understanding mental health.

## Limitations

It is important to also acknowledge potential confounds in PAC research. Specifically, the susceptibility of PAC measures to spurious identification of PAC has recently been highlighted (Aru et al., 2015). Numerous measures have been developed, each with strengths and weaknesses (Hülsemann et al., 2019). In particular higher harmonics from non-sinusoidal cortical activity can result in PAC that does not reflect true neuronal activity. This potential for spurious PAC does not preclude the significance of true PAC, rather it highlights the importance of deliberate methods and analyses in future research to ensure that PAC findings reflect true neural phenomena. Aru et al. (2015) and Jensen et al. (2017) outline key recommendations and considerations for avoiding confounds, including critically the presence of oscillations with clear peaks in a time-resolved power spectrum. A number of advanced signal-processing tools are being developed, including the Extended Modulation Index Toolbox by Jurkiewicz et al. (2020) designed specifically to address these issues.

## Conclusion and Future Directions

Research increasingly suggests that PAC is a fundamental component of human information processing, involved across a range of cognitive processes. Recently, PAC has been implicated in mental health as well, with a number of studies identifying abnormalities in PAC as features of psychotic disorders and ADHD. Decreased symptoms of depression after TMS were also associated with an increase in PAC. More in-depth research is required to fully understand PAC’s role in these disorders, as well as similar research investigating whether abnormalities in PAC are implicated across a range of other mental disorders. However, PAC is believed to be closely associated with neuroplasticity and thus likely critical across mental disorder and treatment. Current findings suggest that better understanding PAC could potentially lead to a better understanding of mental health. While our knowledge regarding PAC is growing, the majority of research to date has focused on cross-sectional adult samples. As much of the initial PAC literature focused on task-based cognitive research, some initial perspectives suggested that PAC is useful only as a transient, state-based measure. However, it is now evident that PAC has both state-based and stable components, considering current evidence demonstrating PAC’s association with mental health at rest and changes over time pre- and post-TMS treatment.

Further research is required to understand PAC during adolescence—a critical period for mental health in which early identification and treatment has been identified as a key strategy for reducing the burden of mental disorder. Longitudinal research focusing on PAC throughout adolescence could help pinpoint the development of psychopathology and the links between cognition and mental health during this period. Combining EEG PAC analysis with DTI for concurrent assessment of structural and functional connectivity may provide the first insights into the relationship between WM microstructure and PAC. For example, coupling between the phase of theta oscillations in prefrontal cortex to the amplitude of gamma oscillations in posterior visual cortex may correspond to increased structural connectivity between these regions. Such research may lead to a greater understanding of the relationship between structural and functional mechanisms underlying information processing and mental health throughout adolescent development. Considering the recently discovered association between PAC and contemporary treatment methods such as TMS, such research has the potential to facilitate a better understanding of the mechanisms underlying such treatment and lead to improved application of these treatments for mental health disorders in the future.

## Author Contributions

All authors listed have made a substantial, direct and intellectual contribution to the work, and approved it for publication.

## Conflict of Interest

The authors declare that the research was conducted in the absence of any commercial or financial relationships that could be construed as a potential conflict of interest.
